# Variable Ophthalmologic Phenotypes Associated with Biallelic Loss-of-Function Variants in *POMGNT1*

**DOI:** 10.3390/ijms26073278

**Published:** 2025-04-01

**Authors:** Lucia Ziccardi, Lucilla Barbano, Mattia D’Andrea, Alessandro Bruselles, Carmen Dell’Aquila, Marcello Niceta, Cecilia Mancini, Alessandro Leone, Mattia Carvetta, Maria Albanese, Emilia Stellacci, Marco Tartaglia, Viviana Cordeddu

**Affiliations:** 1 Clinical and Research Center of Neurophthalmology and Genetic and Rare Diseases of the Eye, IRCCS-Fondazione Bietti, 00198 Rome, Italy; lucia.ziccardi@fondazionebietti.it (L.Z.); lucilla.barbano@fondazionebietti.it (L.B.); carmendellaquila92@gmail.com (C.D.); 2 Department of Medicine and Health Sciences “V. Tiberio”, University of Molise, 86100 Campobasso, Italy; 3 Department of Sense Organs, Faculty of Medicine and Dentistry, Sapienza University of Rome, 00161 Rome, Italy; 4 Department of Oncology and Molecular Medicine, Istituto Superiore di Sanità, 00161 Rome, Italy; alessandro.bruselles@iss.it (A.B.); aleleo1996@gmail.com (A.L.); emilia.stellacci@iss.it (E.S.); 5 Molecular Genetics and Functional Genomics, Bambino Gesù Children’s Hospital, IRCCS, 00146 Rome, Italy; marcello.niceta@opbg.net (M.N.); cecilia.mancini@opbg.net (C.M.); mattia.carvetta@opbg.net (M.C.); marco.tartaglia@opbg.net (M.T.); 6 Department of Humanities, Law and Economics, Telematic University Leonardo da Vinci, UNIDAV, 66010 Torrevecchia Teatina, Italy; 7 Department of Biochemical Sciences “Alessandro Rossi Fanelli”, Sapienza University of Rome, 00185 Rome, Italy; 8 Department of Systems Medicine, University of Rome Tor Vergata, 00133 Rome, Italy; maria.albanese@hotmail.it

**Keywords:** O-linked mannose, α-dystroglycanopathies, *POMGNT1*, whole exome sequencing, retinitis pigmentosa, muscle–eye–brain disease

## Abstract

O-mannosylation is a post-translational modification required for the proper function of various proteins and critical for development and growth. *POMGNT1* encodes the enzyme O-linked-mannose β-1,2-N-acetylglucosaminyltransferase 1, which catalyzes the second step in the synthesis of α-dystroglycan O-mannosyl glycans. Among *POMGNT1*-related α-dystroglycanopathies, muscle–eye–brain (MEB) disease presents with congenital muscular dystrophy, structural brain abnormalities, and retinal dystrophy. Defects in protein O-mannosylation due to biallelic loss-of-function *POMGNT1* mutations produce disturbances in assembling and organizing the basal membrane in the neuroretinal system, involving both the central and peripheral nervous systems. In the retina, POMGNT1 is expressed in photoreceptors and is localized near the photoreceptor *cilium* basal body, a structure critical for protein transport. Recent studies have reported an isolated degenerative ocular phenotype without any involvement of muscular or neuronal tissues. Here, we report on a family with three siblings affected by an apparently isolated clinically variable retinal disease and sharing biallelic inactivating *POMGNT1* variants. Notably, the rod-cone dystrophy phenotype in the three siblings varied significantly in onset, presentation, and severity. These findings provide further evidence of the clinical variability associated with defective POMGNT1 function.

## 1. Introduction

O-mannosylation glycosylation (O-mannosylation) is an essential post-translational modification required for proper functioning of glycoproteins with a role in various developmental and physiologic processes [1]. It occurs in the endoplasmic reticulum via the transfer of mannose from dolichol monophosphate-activated mannose to serine/threonine residues of substrates [2].

Alpha-dystroglycan (α-DG) is the most widely studied O-mannosylated mammalian protein. DGs are glycoproteins that serve as a key component of the dystrophin-glycoprotein complex. This transmembrane multimeric complex consists of dystrophins, sarcoglycans, sarcospan, syntrophins, and several other associated proteins. It is crucial for linking the cytoskeleton of muscle and nerve cells to the extracellular matrix. Indeed, these proteins are essential for maintaining the structural integrity of muscle tissue and are involved in central nervous system development, structural organization and function, myelination, peripheral nerve architecture, epithelial morphogenesis, cell adhesion, and signal transduction [1].

Defective O-mannosylation can lead to a wide range of clinical phenotypes, which emphasizes the various processes finely controlled by O-mannosylated proteins and diverse consequences resulting from their aberrant or impaired function [2]. In particular, inactivating variants in genes encoding glycosyltransferases catalyzing O-mannosylation of α-DG are known as the cause of various congenital muscular dystrophies [2].

*POMGNT1* encodes the protein O-linked-mannose β-1,2-N-acetylglucosaminyltransferase 1, which catalyzes the second step in the synthesis of α-DG O-mannosyl glycans (cores M1 and M2) by covalently attaching a unit of N-acetylglucosamine to the previously added O-mannose residue through a β1–2 linkage [3,4]. *POMGNT1* is one of the 18 genes implicated, when mutated, in the pathogenesis of α-dystroglycanopathies (α-DGPs), a family of disorders including a clinically heterogeneous group of muscular dystrophies with autosomal recessive inheritance caused by glycosylation defect (e.g., muscular dystrophy-dystroglycanopathy (congenital with brain and eye anomalies) type A (MDDGA1 [MIM#236670], MDDGA2 [MIM#613150], MDDGA3 [MIM#253280], MDDGA4 [MIM#253800], MDDGA5 [MIM# 613153], MDDGA6 [MIM#613154], MDDGA7 [MIM# 614643], MDDGA8 [MIM#614830], MDDGA9 [MIM#616538], MDDGA10 [MIM#615041], MDDGA11 [MIM#615181], MDDGA12 [MIM# 615249], MDDGA13 [MIM#615287], and MDDGA14 [MIM#615350]) [5].

Defects in protein O-mannosylation due to loss-of-function (LoF) *POMGNT1* variants also produce disturbances in assembling and organizing the basal membrane in the neuroretinal system [1], as seen in typical characteristics of muscle–eye–brain disease (MEB), which is mainly characterized by the co-occurrence of myopathy, structural brain abnormalities, and retinal dystrophy. POMGNT1, in fact, is expressed also in photoreceptor cells, being localized near the photoreceptor *cilium* basal body, a subcellular structure critical for protein transport from the inner segment to the outer segment of photoreceptors [6]. Notably, biallelic variants in *POMGNT1* have also been reported to underline a rare non-syndromic form of retinitis pigmentosa (RP) (RP76, MIM #617123), highlighting an apparent phenotypic expansion of the *POMGNT1*-related disorders [6,7,8].

RP constitutes a wide group of rare inherited retinal disorders that are characterized by progressive degeneration of rod and, successively, cone photoreceptors, inducing poor night vision, poor peripheral vision, and, finally, central vision loss [9].

In this report, we present a family with three affected siblings presenting with an isolated, autosomal recessive form of rod-cone dystrophy with substantial phenotypic variability, due to the occurrence of compound heterozygosity for two LoF variants in *POMGNT1*.

## 2. Results

### 2.1. Clinical Findings

**Figure 1:**
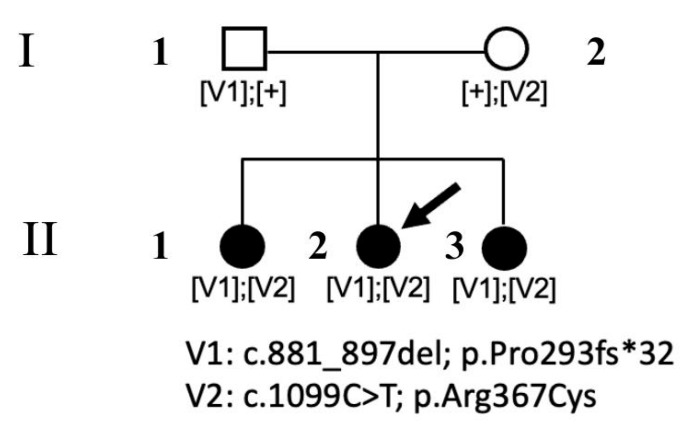
Family pedigree chart. Shaded circles represent affected sisters with non-syndromic rod-cone dystrophy who shared compound heterozygosity for two LoF variants in *POMGNT1*. The black arrow indicates the proband.

The participants in the present study were three sisters (II.1, II.2, and II.3) and their unrelated parents (I.1 and I.2). Figure 1 shows the family pedigree.

**Figure 2:**
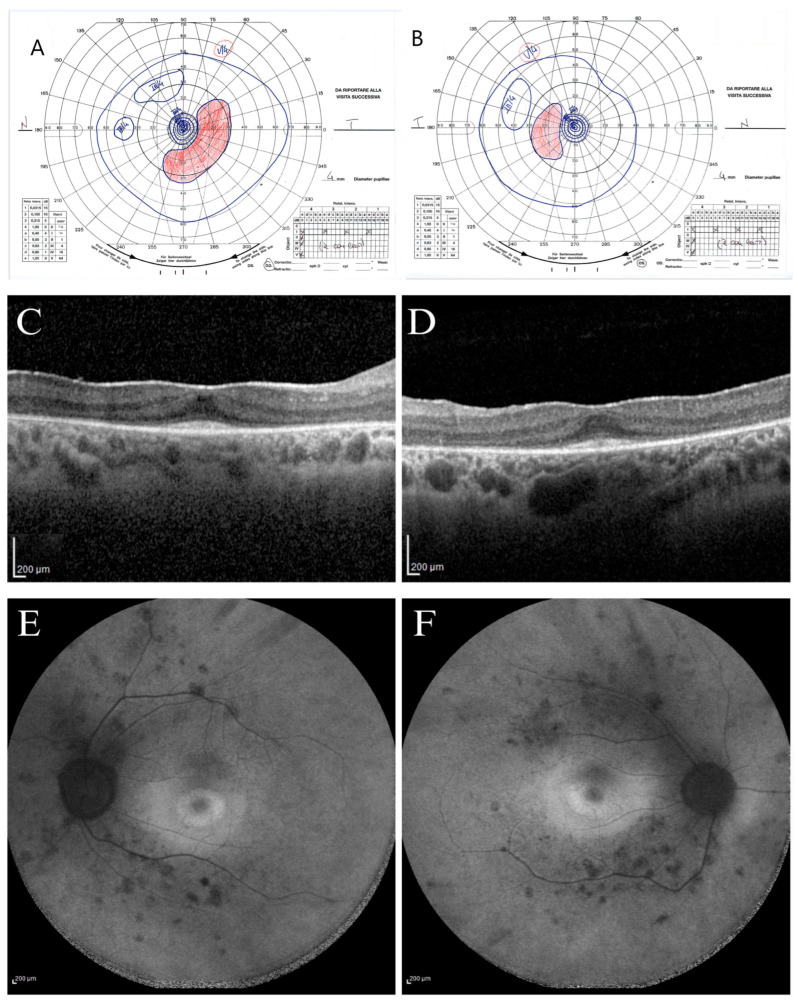
Ophthalmological instrumental assessment of the proband (II.2). The kinetic visual field (VF) of the right (**A**) and left eye (**B**) shows concentrical constriction of all isopters (from I/1 to III/4), with increased blind spot and inferotemporal scotoma in the right eye (**A**). Spectral-domain optical coherence tomography (SD-OCT) scans of right (**C**) and left eye (**D**) show hyper-reflectivity of internal limiting membrane due to an adherent epiretinal membrane, disruption of parafoveal ellipsoid zone, which is preserved only in the foveal region, with an increased subfoveal reflectivity of the basal membrane and retinal pigmented epithelium. Fundus autofluorescence (FAF) shows, in the right (**E**) and left (**F**) eyes, a hyper-autofluorescence ring within the macular region with a parafoveal hypo-autofluorescence demarcations external ring and hypo-autofluorescent spots in the mid periphery, indicative of outer retinal atrophy.

The proband (subject II.2) was a 30-year-old woman followed for photophobia annually since 2013. Her ophthalmological condition was almost stable between 2013 and 2024. At the first visit (2013), the BCVA was 20/20 in both eyes (OU) [correction was of −2.50 cyl axis 10° in her right eye (RE) and of +0.50 sph −2.50 cyl axis 175° in her left eye (LE)], and she could see the smallest character (J1) for near in OU. The chromatic test was normal at all examinations since the Ishihara plates were 22/22 in OU, as was the intraocular pressure (16 mmHg) in OU. The anterior segment examination appeared within normal limits, and only in 2024 did she present with an initial posterior polar cataract in OU. During the initial fundus examination, a slightly pale optic nerve with a globous aspect, thin vessels, ad dystrophic retinal aspect like salt-and-pepper appearance without pigmentary dispersion were observed in OU. Since 2013, a constriction of the visual field detected with the III/4 target with an increased blind spot was observed at the Goldmann perimetry exam. During the 2024 examination, she presented with a slight further constriction of the peripheral isopters (Figure 2A,B).

Since 2013, the light-adapted and the dark-adapted full-field ERGs showed outer retinal dysfunction for both cone and rod components of the whole retina. Indeed, light-adapted and dark-adapted ERG reduced b-wave amplitude and increased b-wave implicit time were found. In 2024, SD-OCT scans showed a disruption of parafoveal and peripheral ellipsoid zone (EZ) with an increased subfoveal reflectivity of basal membrane and retinal pigmented epithelium (RPE). The EZ became not visible outside the parafoveal area toward the retinal periphery; in fact, a preserved EZ was imaged only in the subfoveal area, and an adherent epiretinal membrane (ERM) was found (Figure 2C,D). The FAF showed a stable central hypo-autofluorescence ring surrounded by a hypo-autofluorescence demarcations ring, and hypo-autofluorescent spots were found in the mid periphery along the temporal vascular arcades (Figure 2E,F). During the eleven years (2013 to 2024), patient II.2 showed stable BCVA for near and far in OU, stable chromatic tests, and substantially stable ERG and OCT results, with a slight progression only of peripheral RPE dystrophy and kinetic visual field constriction. At the last visit on July 2024, the patient presented a visual acuity of 20/20 in OU [correction of −0.25 sph −2.50 cyl axis 5° in her RE and −2.25 cyl axis 180° in her LE] and J1 for near in OU. The fundus examination showed a salt-and-pepper dystrophic retinal appearance without massive pigmentary dispersion and thin vessels in OU.

The patient reported fatigue in the lower limbs after walking since childhood, with pain affecting mainly the knees and ankles. In 2020, a neurological examination revealed no deficit of sensibility in the face and limbs, lively and symmetrical osteo-tendon reflexes, and no lack of strength in the limbs. The electroencephalogram showed sporadic slow-cuspid alterations expressed on the bilateral fronto-centro-temporal regions, with a slight right prevalence. Minimal abnormalities in the left dorsal interosseous muscle and right and left tibialis anterior muscles were found using electromyography. In 2023 and 2024, brain and pituitary gland MRI with and without contrast agent showed a slight asymmetry of the adenohypophysis with right sub-sellar floor depression; dynamic imaging did not reveal areas of delayed post-contrast enhancement suggestive of an adenoma. No other general clinical symptoms were reported by the patient.

The proband’s younger sister (participant II.3) was a 20-year-old woman followed since 2013. At the first evaluation (10 years of age), she was asymptomatic, with an ophthalmological evaluation within normal limits. Her BCVA was 20/20 in OU without correction and J1 character for near vision; the anterior segment and the fundus examination appeared within normal limits. After three years, a new evaluation of the patient showed a stable BCVA in OU; however, a slight reduction in the chromatic test was found (21/22 in RE and 19/22 in LE). The kinetic visual field was reliable and appeared within normal limits in OU as well as the dark-adapted and light-adapted ERG responses.

**Figure 3:**
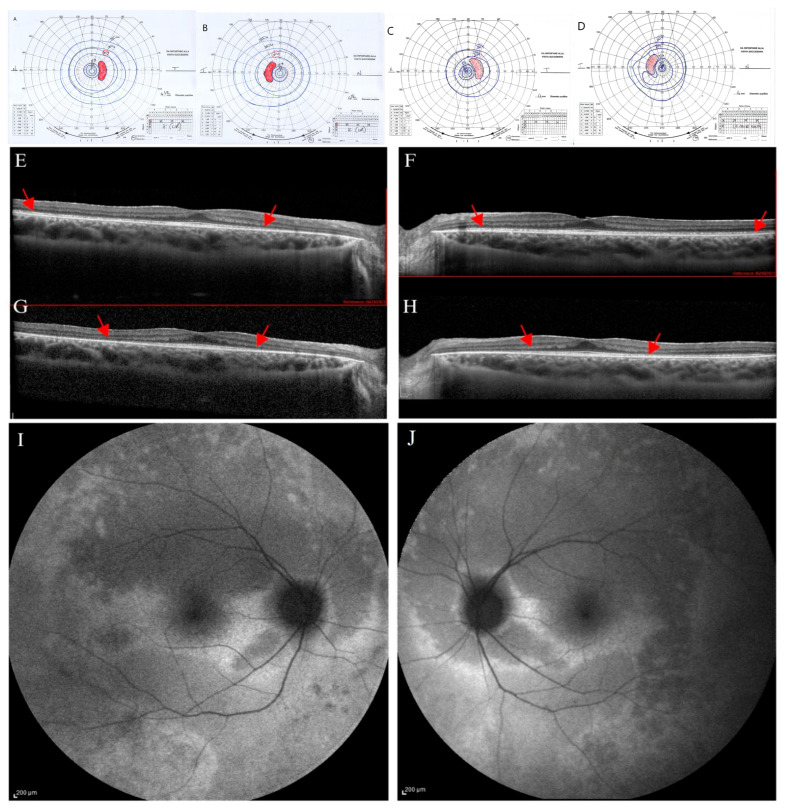
Ophthalmological follow-up (2021–2024) of the younger proband’s sister (II.3). The kinetic visual field (VF) of the right (**A**) and left eye (**B**) performed in 2021 show a peripheral narrowing (isopters I/1 to III/4) with sparing of the central area. Progression of the peripheral constriction is shown in the kinetic VF of right (**C**) and left eye (**D**) performed in 2024. Spectral-domain optical coherence tomography (SD-OCT) scans of right (**E**) and left eye (**F**) performed in 2021 show a focal hyperreflectivity of the internal limiting membrane. Ellipsoid zone (EZ) appears disrupted peripherally and mainly in the nasal parafoveal area (interpapillo-macular region). SD-OCT scans performed in 2024 show a progression of parafoveal EZ disruption in right (**G**) and left eye (**H**), as indicated by the red arrows. Fundus autofluorescence (FAF) shows, in right (**I**) and left eye (**J**), patchy hyper-autofluorescent areas more evident in the inferior sectors and a hyper-autofluorescent area inferior to the macula only in RE (**I**).

At 17 years, genetic testing was performed, and she was re-tested ophthalmologically. At this examination, BVCA was of 20/20 in OU, with a correction of −1.75 sph −2 cyl axis 15° and J1 character for near vision in RE, and of −2 ph −1.50 cyl axis 165° and J1 in LE. The chromatic test was abnormal (15/22 plates in RE and 18/22 plates in LE). The slit lamp biomicroscopy showed an initial posterior lens opacity in OU. The fundus examination showed slight signs of retinal dystrophy in the inferior sector of the retinal periphery, without pigmentary dispersion, in OU, with the macula and retinal vessels within normal limits. The kinetic visual field showed a slight peripheral narrowing with sparing of the central area in OU (Figure 3A,B).

Dark-adapted and light-adapted full-field ERG showed a reduced b-wave amplitude, and mfERG recordings showed reduced function from the foveal center up to the 10 degrees of foveal eccentricity, depicting a progressive bilateral cone-rod dystrophy. SD-OCT scans showed in OU a preserved retinal profile with a normal foveal shape (Figure 3E,F). Inner and outer retinal layers were preserved overall in terms of reflectivity and morphology in the foveal region. EZ appeared disrupted peripherally and mainly in the nasal parafoveal area (interpapillo-macular region). In OU, FAF showed patchy hyper-autofluorescent areas more evident in the inferior sectors and a hyper-autofluorescent area inferior to the macula only in RE (Figure 3I,J).

Over the years (2024), the patient reported the onset of photophobia, along with a stationary BCVA, chromatic test results, and mfERG; progression of the visual field constriction (Figure 3C,D) and SD-OCT EZ parafoveal disruption was observed (Figure 3G,H).

No neurological or muscular symptoms were reported by the patient until adulthood. The neuromuscular examination executed at the age of 16 years was within normal limits. The EMG executed in 2024 did show no abnormalities.

**Figure 4:**
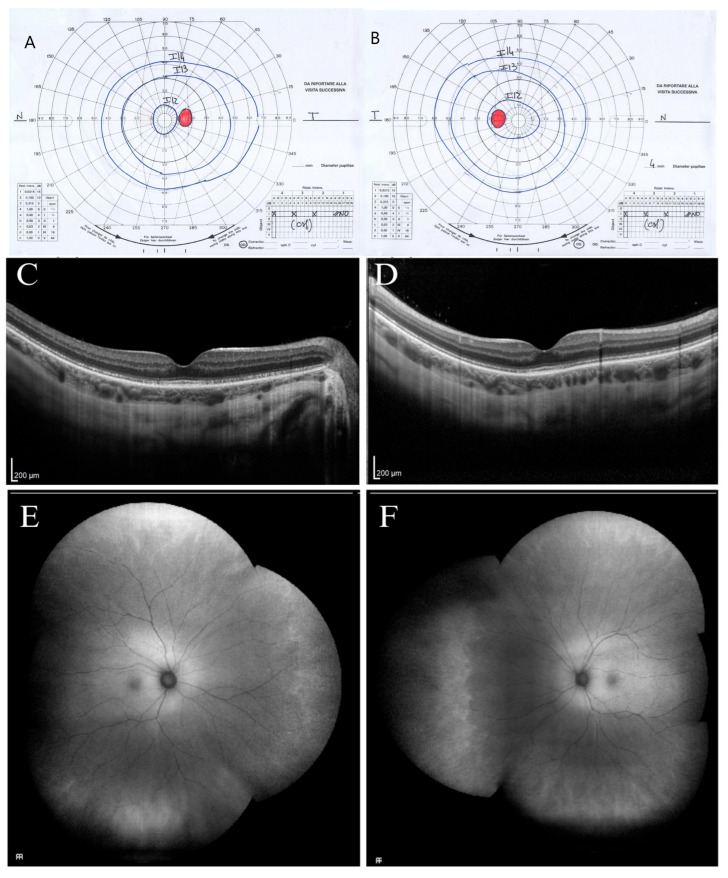
Ophthalmological instrumental assessment of the older proband’s sister (II.1) performed at the last clinical follow-up in 2024. The kinetic visual field (VF) extension of right (**A**) and left eye (**B**) is within normal limits. Spectral domain optical coherence tomography (SD-OCT) scans of the right (**C**) and left eye (**D**) show a peripheral ellipsoid zone disruption involving both extra-foveal and vascular arcades areas. Fundus autofluorescence (FAF) of the right (**E**) and left eye (**F**) is within normal limits.

Participant II.1 was a 32-year-old woman, the older sister of participants II.2 and II.3, who was tested for familial screening. She reported myopia but no photophobia or disturbances in light and dark adaptation or visual field abnormalities. In 2021, BVCA was 20/20 in the RE (−6.50 sph −4.00 cyl axis 170°) and 20/20 in the LE (−4.50 sph −2.75 cyl axis 175°) and J1 character for near vision. The chromatic test, visual field exam, dark-adapted and light-adapted full-field ERG, and FAF were normal. SD-OCT showed a slight extra-foveal EZ disruption. In 2024, the visual field test was within normal limits (Figure 4A,B), and the patient was retested by retinal imaging technology.

Normal retinal UWF-FAF was observed (Figure 4E,F); however, diffuse SD-OCT progressive peripheral EZ disruption involving both extra-foveal and vascular arcades areas were found (Figure 4C,D).

The patient did not report any neurological or muscular symptoms, and the neurological clinical examination was within normal limits.

Subject I.2 was a 53 years-old woman, mother of subjects II.1, II.2, and II.3. She did not refer to any ophthalmological and neuromuscular symptoms. She was tested in 2021, and the BCVA was 20/20 without corrective lenses and J1 character for near vision in OU. Anterior segment and fundus examination showed results within normal limits. Also, the chromatic test, visual field exam, ERG recordings, SD-OCT, and FAF scans were normal. For general health, she reported hypothyroidism and factor V Leiden mutation.

Participant I.1 was a 57-year-old man, the father of participants II.1, II.2, and II.3. He did not report any ophthalmological or neuromuscular symptoms. He was tested in 2021, and the BCVA was 20/20 in OU without corrective lenses and J1 character for near vision in OU. Anterior segment and fundus examination results were within normal limits. Also, the chromatic test, visual field exam, ERG recordings, SD-OCT, and FAF scans were normal. For general health, he reported benign prostatic hyperplasia and blood hypertension.

### 2.2. Molecular Analyses

**Figure 5:**
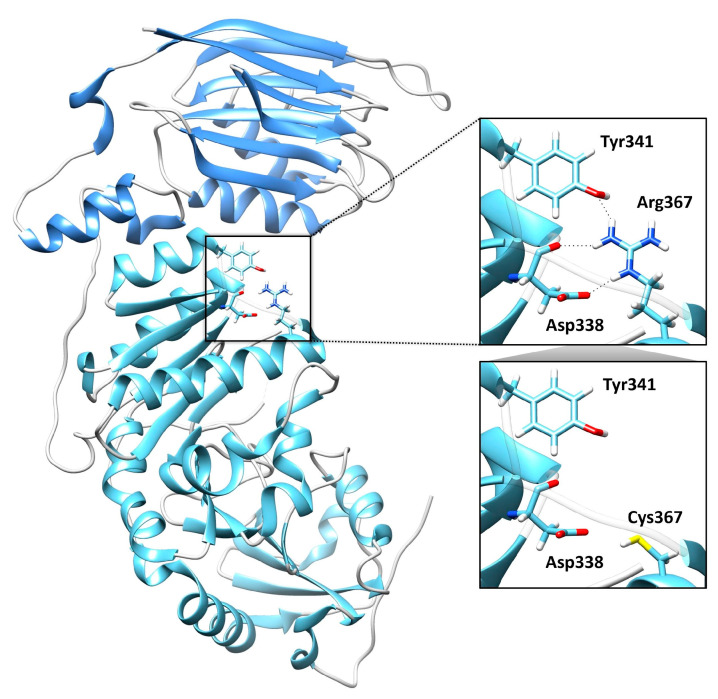
Structural model of the catalytic domain of human POMGNT1 encoded by the *POMGNT1* gene and consequences of the Arg367Cys substitution. On the left, the catalytic domain of POMGNT1 between residues 97–298 is colored with secondary structures and loops in cornflower blue and grey, respectively, whereas the region between residues 299–646, the minimal catalytic domain, is colored with secondary structures in sky blue. On the right, the non-covalent intramolecular interactions (black dashed lines) established by Arg367 with Asp338 (shown both in ribbon and in full stick representation for clarity) and Tyr341 are illustrated (top panel). The replacement of the arginine residue by cysteine abolishes such bonds (bottom panel). All the amino acid side chains retain the color of the respective protein domain, with color-coded non-carbon atoms (white = hydrogen; blue = nitrogen; red = oxygen; yellow = sulfur).

WES revealed two rare/private compound heterozygous *POMGNT1* variants (c.1099C>T, and c.881_897delTCCCAGACAACAAGGTC; NM_017739.4), which were shared by the proband and the two siblings; these were the only putative clinically and functionally relevant events causally associated with the trait (Table 1). Sanger sequencing was performed for variant validation and segregation. The missense variant predicting the p.Arg367Cys substitution is a rare change (dbSNP, rs36038536; MAF = 0.00003966) that was classified as likely pathogenic (LP) according to ACMG criteria. This missense change has been reported in individuals with POMGNT1-related conditions (ClinVar, RCV000648205.8; RCV001835049.1). AlphaMissense Pathogenicity provided a score of >0.995. The variant affects a highly conserved residue within the catalytic domain of the protein [10]. Based on the available POMGNT1 structure (PDB ID: 5GGF), the Arg367Cys amino acid change was predicted to affect the non-covalent intramolecular interactions involving Arg367 with Asp338 and Tyr341 (Figure 5). The frameshift variant was predicted to cause premature truncation (p.Pro293fs*32) and had not previously been reported in public databases. Considering the location of the frameshift (exon 9 of 22), the mutated transcript is expected to undergo nonsense mediated decay.

Based on these findings, we clinically revaluated the three sisters to rule out the occurrence of muscular or neurological features compatible with the clinical presentation of MEB, which is typically associated with biallelic inactivating *POMGNT1* variants.

No involvement of other organs and systems was observed, confirming that the compound heterozygosity for the two *POMGNT1* mutations was causally associated with non-syndromic inherited dystrophy in this family.

## 3. Discussion

Here, we describe three affected siblings showing biallelic LoF *POMGNT1* variants, sharing non-syndromic retinal dystrophy. In the three affected members, the clinical ocular phenotype was variable in age of onset and presented with a wide spectrum of clinical features, from salt-and-pepper appearance (II.2) to retinal dystrophy without pigment dispersion (II.3) to peripheral disruption of EZ only (II.1), with differing severity depending on age.

The proband (II.2) had suffered from retinal dystrophy since she was 19 years. Her phenotype was characterized by a progressive salt-and-pepper retinal degeneration observed throughout the 11-year follow-up, with prevalent dysfunction of rods (rod-cone dystrophy), a progressive constriction of the visual field, and disruption of perifoveal and peripheral EZ on SD-OCT scans. Overall, she did not show neuro-muscular clinical signs, as the first EMG presented with minimal abnormalities not sufficient for a diagnosis of myopathy and not confirmed at the last examination. Neuro-radiological scans were also within normal limits, except for the adenohypophysis.

The proband’s younger sister (II.3) was negative for ocular signs and symptoms until the age of 10 years. At the age of 17 years, when the genotype was known, she was carefully re-observed, and she showed initial signs of peripheral retinal dystrophy as well as peripheral narrowing during the VF examination, retinal and macular dysfunction (ff-ERG and mfERG), and disrupted peripheral EZ at SD-OCT. A progressive retinal phenotype was observed over the following 3 years (up to the age of 20 years). However, the patient tested negative for all neuromuscular conditions, like the proband.

Surprisingly, the proband’s older sister (II.1), who never complained of visual symptoms and was asymptomatic for photophobia, dark-adaptation, as well as visual field abnormalities until 2021, also started to show, in 2024, minimal signs in retinal dystrophy, consisting of a subtle EZ disruption involving both extra-foveal and vascular arcades areas, but with VF and ff-ERG examinations within normal limits. The patient (II.1) did not report any neurological or muscular symptoms throughout the follow-up period and had a negative neurological exam.

Previous reports have already described non-syndromic RP associated with *POMGNT1* gene mutations. Wang et al. [7] reported 17 patients from five families affected by RP, with an onset between the ages of 12 and 40 years, recurring in autosomal recessive inheritance without other systemic manifestations, with no intellectual disability and no muscle weakness or atrophy. The clinical features of the participants were a typical RP with attenuated retinal arterioles, bone spicule pigment deposits, optic disc pallor, severely reduced rod responses on ERG examination. Similarly, Xu et al. [6] described typical clinical and morphological features of RP in four patients from three unrelated families with *POMGNT1* gene mutations without extraocular involvement. Besides providing further evidence of isolated retinal involvement, the present findings document intrafamilial clinical variability of the retinal disease presentation with different retinal morphological and functional features in the three examined patients, characterized by aspect of either diffuse salt-and-pepper dystrophy or retinal dystrophy without pigmentary dispersion or peripheral disruption of EZ only. Using SD-OCT, the EZ disruption was observed from the perifovea to the retinal periphery in participants II.2 and II.3, whereas it appeared as an extra-foveal EZ rarefaction in participant II.1.

In agreement with Patel et al. [8], we observed that the clinical severity was independent of the patients’ age; in fact, the slightest signs of retinal dystrophy were observed in the older sister in our cohort. Moreover, Patel et al. [8] also found phenotypic heterogeneity of the ocular phenotype from retinal dystrophy to uveitis to neuro-ophthalmological presentation.

It has been proposed that the isolated occurrence of retinal disease in these families is likely linked to the occurrence of residual POMGNT1 catalytic activity [6]. As shown for other genes implicated in dystroglycanopathies [11,12], it has already been suggested [10] that the severity of the disease correlates with the strength of the disruptive effect of *POMGNT1* mutations. The significantly milder non-syndromic phenotype appears to be consistent with the missense mutations assessed as having residual catalytic activity. However, the role of other contributing factors or more complex genetics cannot be ruled out *a priori* [13]. Based on the few reported cases of *POMGNT1*-related dystrophies, it is also unknown whether epigenetic and environmental factors or lifestyle habits can play a role in the presentation, severity, and progression of this disease entity.

In recent years, WES has been successfully used to identify the molecular bases of rare phenotypic patterns. In this case, we addressed this unsolved family using WES analysis after observing that the proband was affected by sporadic isolated retinal dystrophy and apparently negative for mutations in IRD-associated genes that had been assessed using an NGS gene panel. Following the identification of the biallelic *POMGNT1* variants in the proband and the two sisters, clinical reassessment allowed us to confirm the occurrence of a retinal phenotype in the younger and older siblings The clinical re-evaluation of the three affected individuals excluded major neurological and neuromuscular involvement, thus excluding a syndromic phenotype.

The present findings, together with the previous reports by Wang et al. [7], Xu et al. [6], and Patel et al. [8], provide evidence that the *POMGNT1* gene should be included in the genetic panels for isolated IRD. Our results highlight the importance of broad-spectrum genetic screening in both isolated and familial cases that remain unresolved with conventional targeted gene panels associated with hereditary retinal dystrophies. Indeed, by investigating genes associated with syndromic forms, it is possible to diagnose conditions linked to these genes, even when the phenotype is limited to ocular manifestations, with little or no systemic involvement or with minimal, subclinical alterations in other organ systems.

The identified p.Arg367Cys change is a rare substitution affecting a conserved arginine within the catalytic domain. The residue is annotated as intolerant to changes according to Metadome predictor (tolerance score = 0.39; intolerant) and AlphaFold DB prediction (AlphaMissense Pathogenicity score = 0.985) tools. Notably, a different disease-causing substitution affecting the same residue (p.Arg367His) has previously been reported in a patient with less severe congenital muscular dystrophy, dyspraxia, feeding difficulties, and seizures but with no weakness or delay of acquired ambulation [14]. Taking into account that the frameshift variant is predicted to cause premature protein truncation (p.Pro293fs*32) and nonsense-mediated decay of the transcript, the pathogenetic mechanism underlying the isolated retinal phenotype is likely associated with the occurrence of residual POMGNT1 function. Of note, the three members of the family showed variable clinical features and disease onset, which might indicate that other genetic, epigenetic, or environmental factors can contribute to the expressivity of the condition. Additional clinical reports are required to more accurately characterize the clinical spectrum and the underlying contributing causes of *POMGNT1*-related dystrophies.

Moreover, the *POMGNT1* gene has a broad range of expression variability within the members of the same family, as our case description also depicts.

This aspect is shared with many other inherited retinal degenerations among the same family members [15]. The present report demonstrates the need to extend genetic testing to apparently unaffected family members in order to identify even subtle pathological conditions and initiate early follow-up for these individuals. The clinical diagnosis in the younger proband’s sister, almost contemporary to the WES results, is a perfect example of the crucial role of the clinical follow-up. Moreover, the identification of slight clinical abnormalities in the older proband’s sister points to the sub-clinical presentation of genetic diseases, which are difficult to identify when the genotype is still not resolved and the patient is asymptomatic. Based on the obvious clinical presentation of retinal degeneration phenotype in the proband and ignoring the subclinical signs of the ocular disease in other two sisters, who were asymptomatic in the first clinical assessments, the pathology could be identified far earlier thanks to high-tech instrumental ophthalmological testing and because molecular tests were extended to all family members. As a result of our considerations, the youngest patient (II.3) received a clinical diagnosis because enrolled in the WES study. The oldest patient (II.1), still presenting subclinical retinal dystrophy, could be considered a pre-symptomatic diagnosis, despite the age.

## 4. Conclusions

Our findings underscore the importance of comprehensive genetic screening in both isolated and familial cases of retinal degeneration, particularly in instances where conventional panels of non-syndromic retinal dystrophies fail to yield a diagnosis. The identification of biallelic *POMGNT1* variants through WES in a family with an extremely different retinal phenotype and without neuromuscular involvement highlights the variable phenotypic expression of mutations in *POMGNT1*. The description of an additional cohort of patients with non-syndromic retinal dystrophy related to *POMGNT1*, traditionally causative of MEB disease, emphasizes the need for broader genetic testing, especially in patients with verified isolated retinal dystrophy and nonconclusive genotype. Notably, the clinical variability observed between siblings with identical genotype highlights the challenges in predicting disease onset and severity, reinforcing the significance of early and proactive follow-up of family members, even when they are declared asymptomatic. This case description also suggests that timely genetic testing and thorough ocular instrumental evaluations can facilitate early detection of subclinical retinal changes, allowing for more adequate management and avoidance of unnecessary diagnostic interventions. We acknowledge as the main limitation of the present study the small number of enrolled subjects. Further studies are needed to validate the present findings. Overall, this study advocates for a more inclusive approach to genetic based, with the potential to improve diagnostic accuracy and clinical outcomes for individuals with hereditary retinal dystrophies.

## 5. Materials and Methods

Three siblings born to non-consanguineous parents were enrolled in a research program dedicated to undiagnosed patients affected with retinal diseases. The research program was coordinated by the Clinical and Research Center of Neuro-ophthalmology, Genetic and Rare Diseases of the Eye of IRCCS Fondazione Bietti for patient enrollment and sampling. Genomic analyses were coordinated by the Istituto Superiore di Sanità and Ospedale Pediatrico Bambino Gesù, Rome, Italy. Clinical data and DNA samples were collected, stored, and used following the procedures in accordance with the Helsinki Declaration (1964 and further revisions), and the study was approved by the local ethics committee (Comitato Etico Centrale IRCCS Lazio, Sezione IFO/Fondazione Bietti, Rome, Italy).

Upon recruitment in the study (FB RET-02-2019), executed from February to July 2019 at the IRCCS Fondazione Bietti, informed consent after full explanation was obtained from each participant included in the study. Explicit permission was obtained to publish the fundus photographs by the participants involved in the study.

### 5.1. Ophthalmological Assessments

Patients underwent a full ophthalmologic evaluation by slit lamp examination of anterior and posterior chambers as well as ocular tonometry. Accurate functional and morphological assessments were performed. As for functional tests, we performed best corrected visual acuity (BCVA) measured by the Early Treatment Diabetic Retinopathy Study (ETDRS) charts (Lighthouse, Low Vision Products, Long Island City, NY, USA) at a distance of 4 m and expressed as Snellen equivalent, Jaeger Reading Charts at 40 cm for near vision, and a chromatic test by the monocular administration of Ishihara pseudoisochromatic plates (24 plates edition, Kanehara Trading Inc., Tokyo, Japan) in natural daylight. Moreover, patients underwent kinetic Goldmann perimeter (Haag-Streit 940, Kőniz, Switzerland) and dark-adapted and light-adapted full-field electroretinogram (ERG) (Retimax Advanced Plus apparatus, CSO, Firenze, Italy) and multifocal ERG (mfERG) (VERIS Clinic TM version 4.9; Electro-Diagnostic Imaging, San Mateo, CA, USA) according to the 2011 International Society for Clinical Electrophysiology of Vision (ISCEV) using Dawson–Trick–Litzkow (DTL) contact electrodes with the pupil dilated at 8 mm [16].

As for structural retinal assessment, accurate fundus multimodal imaging was assessed by dilated pupil fundus examination using + 90D non-contact lens (Volk Optical, Mentor, OH, USA). Also, patients underwent retinal imaging which was executed by ultrawide field fundus photograph obtained with Optos California (Optos PLC, Dunfermline, Scotland, UK); Fundus Auto Florence (FAF) (488 nm excitation, barrier filter transmitted light from 500 to 680 nm, 55°) and optical coherence tomography (OCT) were acquired using Heidelberg Spectralis OCT (HRA + OCT, Heidelberg Engineering, Heidelberg, Germany). Only in patient II.1, FAF was acquired by Clarus 770 Ultrawide-Field (UWF) system (v1.1; Zeiss AG, Oberkochen, Germany).

### 5.2. Neurological Assessment

Neurological clinical examination, including evaluation of sthenia, tenderness, osteotendinous reflexes, tone, and trophism, was executed at the Neurology Unit of University Hospital Tor Vergata on three participants. Electromyography (Natus EliteSoftware powered by Keypoint) was performed in the proband and the younger sister in 2020 and 2025. In the proband, electroencephalogram (EEG) recording (by 32-channel EBNeuro system, sampling rate 128 Hz) was performed in 2020, and brain magnetic resonance imaging (MRI) (on high-field magnet 1.5 T, Philips Ingenia, Philips Healthcare, Amsterdam, The Netherlands), including diffusion-weighted, T1- and T2-weighted tonograms, and detailed study of the pituitary gland before and after the administration of paramagnetic contrast agent, was performed in 2023 and 2024.

### 5.3. Genomic Analysis

Whole exome sequencing (WES) and data analysis were performed as previously reported [17,18]. In brief, the genomic DNA of the three sisters was extracted from leukocytes and sequenced using Illumina paired-end technology. Libraries were obtained using the SureSelect Human All Exon v.7 kit (Agilent, Santa Clara, CA, USA). WES raw data were processed and analyzed using an in-house implemented pipeline, as previously described [7], according to the GATK’s Best Practices [19]. The UCSC GRCh37/hg19 version of genome assembly was used as a reference for read alignment (BWA-MEM tool) and variant calling (HaplotypeCaller, GATK v3.7) [19,20]. Variants’ functional annotation was performed using the SnpEff v.5.0 and dbNSFP v.4.5 tools [21,22]. Relevant in silico impact prediction tools, such as Combined Annotation Dependent Depletion (CADD) v.1.6, AlphaMissense Pathogenicity Heatmap, Mendelian Clinically Applicable Pathogenicity (M-CAP) v.1.0, and InterVar v.2.0.1, [23,24,25], were also used. By filtering against our population-matched database (>3000 exomes) and public databases (dbSNP152 and gnomAD v.3.1), the analysis focused on high-quality rare variants affecting the coding sequence and adjacent splice site regions. Finally, the ClinVar database (https://www.ncbi.nlm.nih.gov/clinvar, accessed on 20 Jannuary 2025) was adopted for variant interpretations and classification according to ACMG recommendations.

### 5.4. Structural Analysis

The non-covalent intramolecular interactions involving Arg367 in the wild-type (WT) POMGNT1 structure (PDB ID: 5GGF) [26] and the predicted structural consequences of the Arg367Cys amino acid change were inspected using UCSF Chimera software v.1.17.3 (https://www.cgl.ucsf.edu/chimera (accessed on 20 January 2025)) [27].

## Figures and Tables

**Table 1:** WES data output.

WES enrichment kit	Agilent SureSelect Human All Exon V7
Total passing quality filters unique reads	127,841,845
% target regions with coverage >10×	95.5
% target regions with coverage >20×	93.5
Median sequencing depth on target	125×
Private and low frequency variants with functional effect	351 ^1^
Putative disease genes [AR trait]	78 ^2^
Genes with biallelic events shared by all probands	1 ^3^

^1^ Private or low frequency [gnomAD MAF < 0.1% and recurrence <1% within our >3000 WES database] non-synonymous single nucleotide variants or indels within coding exons and splice regions [−3/+8]. ^2^ Functional impact assessed by CADD v.1.6, M-CAP v.1.0 and InterVar v2.0.1. Variants predicted as benign or likely benign by InterVar were discarded, and only those with a CADD score >20 or an M-CAP score >0.025 were retained. ^3^ *POMGNT1* (c.1099C>T, p.Arg367Cys; c.881_897delTCCCAGACAACAAGGTC, p.Pro293fs*32).

## Data Availability

Data are available upon request.

## Abbreviations

The following abbreviations are used in this manuscript:

α-DG	Alpha-Dystroglycan
α-DGPs	A-Dystroglycanopathies
LoF	Loss-Of-Function
MEB	Muscle-Eye-Brain Disease
RP	Retinitis Pigmentosa
BCVA	Best Corrected Visual Acuity
ETDRS	Early Treatment Diabetic Retinopathy Study
mfERG	Multifocal ERG
DTL	Dawson–Trick–Litzkow
FAF	Fundus Auto Florence
SD-OCT	Spectral Domain Optical Coherence Tomography
UWF	Ultrawide-Field
EEG	Electroencephalogram
MRI	Magnetic Resonance Imaging
WES	Whole Exome Sequencing
CADD	Combined Annotation Dependent Depletion
M-CAP	Mendelian Clinically Applicable Pathogenicity
OU	Both Eyes
RE	Right Eye
LE	Left Eye
EZ	Ellipsoid Zone
RPE	Retinal Pigmented Epithelium
ERM	Epiretinal Membrane
VF	Visual Field
EMG	Electromyography
FF	Full Field
